# Animal models in venom and antivenom research: The need to align academic discovery with manufacturing and regulatory expectations

**DOI:** 10.1371/journal.pntd.0014537

**Published:** 2026-07-27

**Authors:** Stuart Ainsworth, Nicholas R. Casewell, Marco Aurélio Sartim, Amy E. Marriott, José María Gutiérrez

**Affiliations:** 1 Department of Infection Biology and Microbiomes, Institute of Infection, Veterinary and Ecological Sciences, University of Liverpool, Liverpool, United Kingdom; 2 Centre for Snakebite Research & Interventions, Liverpool School of Tropical Medicine, Liverpool, United Kingdom; 3 Faculdade de Ciências Farmacêuticas, Universidade Federal do Amazonas, Manaus, Amazonas, Brazil; 4 Fundaçao de Medicina Tropical – Dr Heitor Vieira Dourado, Manaus, Amazonas, Brazil; 5 National Centre for the Replacement, Refinement and Reduction of Animals in Research, London, United Kingdom; 6 Instituto Clodomiro Picado, Facultad de Microbiología, Universidad de Costa Rica, San José, Costa Rica; Fundação de Medicina Tropical Doutor Heitor Vieira Dourado: Fundacao de Medicina Tropical Doutor Heitor Vieira Dourado, BRAZIL

## Abstract

The study of snake venom toxicity and evaluation of the neutralisation ability of antivenoms and novel therapeutic agents for envenoming has largely relied on animal tests, especially the analysis of the lethal activity of venoms and its neutralisation. In addition, other animal-based assays are used to assess pathology-specific effects of venoms. Despite their demonstrated value, these assays have several limitations. They involve acute stress and pain in animals, and their validity, *vis-à-vis* the characteristics of human snakebite envenoming, is limited owing to the nature of the assays. Therefore, urgent innovations are required in this field. Ensuring the implementation of the 3Rs (Replacement, Reduction, and Refinement) is essential when animal studies are required. These include: (a) refinement of the tests (e.g., use of analgesia, reduction of assay duration, and refining of humane endpoints); (b) reduction in the number of animals used by more thorough *in vitro* assessment of antivenoms prior to *in vivo* assays, and improved statistical analyses and group sizing; and, most importantly, and (c) replacement of *in vivo* rodent tests with validated *in vitro* approaches, such as immunoassays, enzymatic and cell-based assays, the use of invertebrate models, and the implementation of New Approach Methodologies (NAMs), i.e., novel *in vitro* or *in silico* techniques that model complex pathophysiological processes of envenoming. A roadmap involving researchers, manufacturers, quality control groups, regulators, and funding agencies is proposed, with the aim of driving significant changes in this field.

## Author summary

The study of toxic activities of venoms and their neutralisation by antivenoms, which are the standard treatment for envenoming, has been largely based on the use of animal models. The most widely used test in this field is the mouse lethality assay, which involves considerable murine stress, pain, and suffering. Moreover, despite its widespread use, there is a growing body of evidence suggesting that this assay does not always reproduce the most relevant human clinical manifestations of envenoming. At a global level, the use of animal models in research and manufacture of drugs is being questioned on ethical and scientific grounds, and the paradigm of the 3Rs (Replacement, Reduction, and Refinement) is being promoted with the aim of improving animal-based tests and, ultimately, of replacing these tests by *in vitro* alternative methods. This review summarises the main tests involving animals used in the field of venom research and antivenom manufacture and quality control, presents a critical view of the limitations of the animal models, and discusses current and future alternatives to replace animal-based assays by *in vitro* methods that correlate with the main toxic effects of venoms. A roadmap to guide these efforts is proposed, with short-, medium-, and long-term goals.

## Introduction

Snakebite envenoming represents a major, yet under-recognised, public health issue globally, particularly in sub-Saharan Africa, Asia, and Latin America, where substantial morbidity and mortality occurs [1]. Since the late 19th century, effective and validated treatment has relied almost exclusively on the administration of antivenoms, which are preparations of antibodies, or antibody fragments, obtained from the plasma of animals immunised with snake venoms [2]. In addition, promising therapeutic alternatives have emerged in recent years, such as recombinant antibodies, and natural and synthetic inhibitors [3,4]. Since the beginnings of antivenom development, the evaluation of their neutralising efficacy has primarily been conducted in animal models at the preclinical level. The importance of preclinical models for evaluating antivenom efficacy should not be understated, as regulatory approval often relies solely on preclinical data to provide accurate assessments of efficacy.

The ‘gold standard’ preclinical model remains the venom lethality assay, which assesses the capacity of an antivenom to neutralise venom-induced lethality, usually in mice [5]. In addition, preclinical assays, both *in vitro* and *in vivo*, have been developed for assessing other toxic effects of venoms [5]. These preclinical models have been widely adopted by academic researchers, antivenom manufacturers, and quality control laboratories. They are also listed in regulatory documents, such as Pharmacopoeias and technical guidelines [5]. Despite the widespread use of animal models in this field, major advances over the past two decades in understanding venom composition and mechanisms of action are enabling novel, 3Rs-aligned tests (Replacement, Reduction and Refinement of animal models), all driven by the shared objective of generating robust evidence to achieve the best outcomes for envenoming patients with reduced reliance on animal testing.

The present review summarises the currently accepted preclinical methods to assess venom toxicity and antivenom potency, mostly based on animal models. The limitations of these methods are discussed, together with an analysis of novel alternatives which more closely represent the main clinical manifestations of envenoming. We then propose a practical implementation roadmap for aligning academic model development, manufacturing, quality control, and regulatory expectations within a 3Rs framework.

## Current WHO-recommended preclinical methods

The World Health Organization (WHO) Guidelines for the Production, Control and Regulation of Snake Antivenom Immunoglobulins, which were adopted in 2008 and updated in 2017 [5], provide methodologies for antivenom manufacture and preclinical assessment of treatment efficacy. The WHO guidelines outline two types of assays: the essential assay, based on the neutralisation of venom lethality, and recommended assays which are pathology-specific.

### The essential assay: Lethality neutralisation (LD_50_/ED_50_)

The venom-induced lethality assay remains the essential preclinical assay to evaluate venom toxicity and antivenom neutralising capacity. In practice, the protocol involves two key steps. Firstly, it is used to establish the lethality of venom in mice (the Median Lethal Dose (LD_50_)), followed by the determination of the antivenom dose, in volume or mass of antivenom, or venom/antivenom ratio, needed to neutralise the lethal effect of venoms [5] (the Median Effective Dose (ED_50_)).

For these assays, the WHO recommends incubating mixtures containing a fixed dose of venom (challenge dose, typically 3–5 x LD_50_s) with variable dilutions of antivenom before injection in mice using the intravenous (i.v.) or intraperitoneal (i.p.) routes. Subsequently, a binary outcome measure of alive or dead is recorded at a predefined timepoint, most commonly 24 h (when using the i.v. route) or 48 h (when using the i.p. route) after dosing. The fixed challenge dose and variable therapy dose format allows the assessment of dose-response relationships to be established by standard statistical approaches (e.g., probits or Spearman-Kaber) based on resulting experimental survival in the different dose groups [5].

Although mice are the most widely used species in preclinical antivenom assessment, different strains may show variable susceptibility and pharmacodynamic responses to venoms [6]. Nevertheless, in antivenom quality control settings, only a limited number of mouse strains are employed to ensure consistency and regulatory compliance [5,7]. Other species, such as rats, rabbits, guinea pigs, or larger mammals, are mainly used for specific research purposes, offering insights into specific pathophysiological mechanisms and pharmacokinetic/pharmacodynamic aspects of envenoming [8].

Despite biological variation in both the model and the test agent (i.e., venom), if well executed, the lethality assay is reproducible and provides comparable measures of venom lethality and the ability of a therapy to neutralise lethal venom effects [9]. However, due to its reliance on pre-incubation and co-delivery and a binary live/dead endpoint, it is substantially limited in its ability to provide information on impacts in treatment delay, toxin-therapy pharmacokinetics and pharmacodynamics, or envenoming pathology-specific clinical outcomes.

### Supplementary assays for specific venom-induced pathologies

In addition to the essential assay of neutralisation of lethality, the WHO guidelines describe a set of supplementary assays designed to quantify specific venom-induced pathological effects and their neutralisation by antivenoms. These supplementary assays are recommended when new antivenoms are developed or existing antivenoms are introduced into new regions, but are not required for routine quality control [5].

#### Haemorrhagic activity.

Snake venom-induced haemorrhage is a common clinical manifestation primarily caused by metalloproteinases (SVMPs) abundant in viperid venoms [1]. These enzymes disrupt the integrity of capillary blood vessels, resulting in bleeding into surrounding tissues. Additionally, SVMPs and other venom components disrupt haemostasis, further exacerbating haemorrhage [10].

The protocol for assessing haemorrhagic activity in animal models relies on the visual measurement of the haemorrhagic halo induced by the intradermal (i.d.) administration of venom. The minimum haemorrhagic dose (MHD) corresponds to the dose of venom that induces a haemorrhagic lesion of 10 mm diameter in the inner side of the skin 2 or 3 h after injection [5].

#### Coagulopathic activity.

Coagulation disturbances are common in envenomings by viperid and some elapid snakes. Coagulopathic venoms contain haemostatically-active toxins, which are procoagulant by activating blood clotting factors, generating a venom-induced consumption coagulopathy (VICC) [11]. This mechanism is typically assessed *in vitro* by measuring clot formation in human plasma or fibrinogen, with the minimum coagulant dose (MCD) defined as the venom concentration inducing clotting within 1 min. This effect is mainly mediated by serine proteinases (SVSPs) that convert fibrinogen into fibrin, and SVMPs which activate factor X or prothrombin [10,11].

However, considering the *in vivo* context in human patients or animals, venom-induced procoagulant effect leads to the consumption of clotting factors resulting in VICC. This leads to incoagulability, with alterations in laboratory clotting tests, an effect that contributes to systemic bleeding disorders [10,11]. This *in vivo* defibrinogenating activity is assessed by determining the ability of venoms to induce incoagulability in rodents, expressed as the minimum defibrinogenating dose (MDD), i.e., the dose of venom that renders blood unclottable 1 h after i.v. injection [5].

#### Necrotising activity.

Tissue necrosis is a major local complication of viperid and some elapid envenomings, often leading to permanent sequelae, resulting from direct damage to cells and extracellular matrix components, or indirect effects secondary to ischaemia and inflammation [12]. In viperids, SVMPs and phospholipases A_2_ (PLA_2_s) directly degrade basement membranes and other extracellular matrix components and cause irreversible cell damage at the various layers of the skin [13]. In some elapids, such as the spitting cobras, cytotoxic PLA_2_s and cytotoxic three-finger toxins (3FTxs) disrupt cell membranes triggering tissue necrosis [14]. The necrotising activity is evaluated by determining the extension of the dermal skin necrosis in mice injected with venom by the i.d. route. The minimum necrotising dose (MND) corresponds to the dose that induces a necrotic lesion of 5 mm diameter after 72 h [5].

#### Myotoxic activity.

Snake venom-induced myotoxicity primarily results from the direct action of PLA_2_s and enzymatically inactive PLA_2_ homologues which disrupt muscle cell plasma membranes by catalytically-dependent or independent mechanisms, respectively, leading to irreversible cell damage [15]. Other venom components, including SVMPs, cytotoxins of the three-finger toxin family, and β-defensin-like myotoxins, can also contribute to muscle injury by different mechanisms. Depending on the venom, muscle damage may occur only locally, at the site of venom injection, or may be systemic, i.e., rhabdomyolysis, which may result in acute kidney injury [15,16].

**Fig 1:**
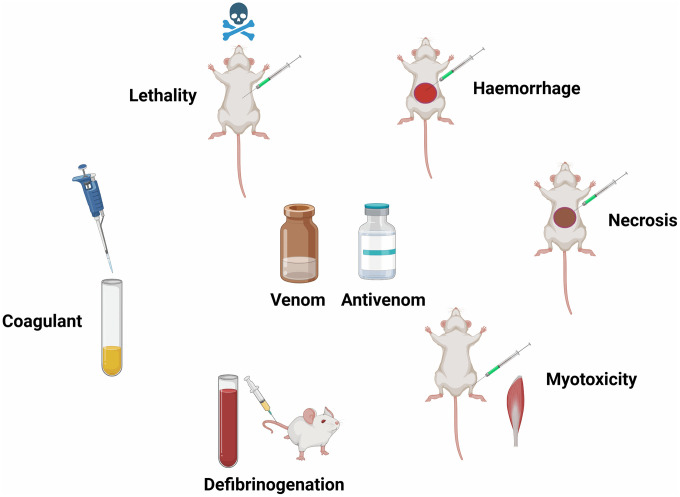
Representation of the assays to assess the various toxic activities of snake venoms and their neutralisation by antivenoms, according to the WHO Guidelines for the Production, Control and Regulation of Snake Antivenom Immunoglobulins [5]. Descriptions of the assays are provided in the text. Created in BioRender. Gutiérrez, J. M. (2026) BioRender.com/8gx68hg.

Experimentally, venom myotoxicity is assessed by the intramuscular injection of venom in mice, followed by the quantification of creatine kinase (CK) activity in plasma 3 h after injection. Since CK is a cytosolic muscle enzyme, plasma CK activity is a well-known and reliable biomarker of skeletal muscle tissue necrosis [5]. Figure 1 summarises the main preclinical tests to assess venom toxicity and neutralisation by antivenoms.

## Practical strengths and limitations of conventional animal models of envenoming

### Strengths

Conventional murine preclinical models of envenoming have underpinned the antivenom field for many decades [5]. Their continued use reflects the strengths of the assays, i.e., their standardisation, ease of implementation and interpretation, but these features also create limitations when the assays are used to infer real world clinical performance and to guide intervention.

The strengths of these preclinical models are considerable. Snake venom contains a mixture of toxins that vary between species, with different toxin classes or isoforms acting in a highly variable manner on distinct physiological systems [17]. The animal lethality model enables simultaneous evaluation of toxin activity, including the potential influence of synergistic or additive effects.

Furthermore, the ED_50_ assay enables a rapid readout of venom neutralisation, with clear experimental outcomes (dead/alive). Thus, these models have been highly informative in: (a) the development and evaluation of specific antivenoms, (b) enabling comparative analysis of products available in certain territories, (c) identifying deficiencies in therapeutic coverage against specific snakes, and (d) exploring appropriate antivenom use in situations where specific products against certain species either do not exist, are not available or have expired [18–21]. Ultimately, the use of these assays over several decades has enabled regulatory decision-making, resulting in the approval of antivenoms for use in snakebite patients in many different settings, which is particularly important given the paucity of data associated with the efficacy of antivenoms from robust clinical trials [22,23].

### Limitations

**Fig 2:**
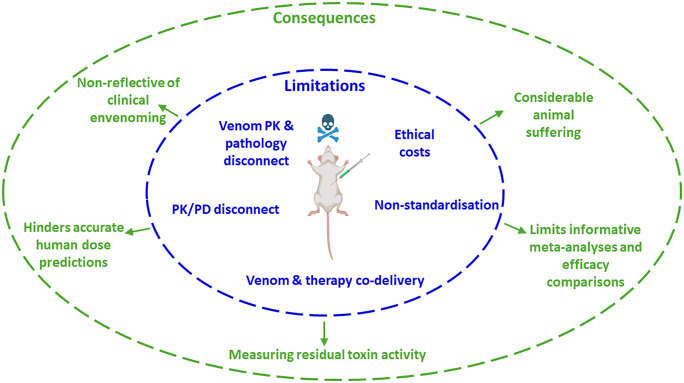
Summary of the limitations, and the consequences of these limitations, of the current animal-based lethality assays to assess venom toxicity and neutralisation of venoms by antivenoms.

Although widely used in envenoming science, rodent lethality assays possess substantial limitations. In addition to the pain and distress involved in these tests, they poorly replicate natural snakebites, while rodent responses may not reflect human envenoming due to physiological differences. Known examples of this include differences in: (a) the affinity of certain α-neurotoxins to human and rodent nicotinic acetylcholine receptors [24], (b) the sensitivity of rodent and human plasma proteins to the effects of procoagulant toxins [25], and (c) human and mouse skin structure, physiology and associated immunology [26], which seem likely to impact on the pathophysiology of local envenoming. These factors restrict translational relevance and increase the risk of equating preclinical results to clinical effectiveness in real world envenoming [27]. Figure 2 summarises some of the key limitations, and the consequences of these limitations, of the current animal models used in venom and antivenom studies, and these are discussed in detail in the following sections.

#### Assay design features are disconnected from the clinical scenario.

The core limitation of the murine lethality assay is that it is a challenge test rather than a treatment simulation. The assay measures the neutralising capacity of a product under a controlled challenge condition. It does not assess clinical rescue, which depends on time-dependent pharmacokinetics and pharmacodynamics. As a result, the pathologies evaluated in mice do not always reflect the pathology which is most relevant in patients. Perhaps the most extreme example of preclinical pathology being disconnected to clinical signs of envenoming relates to the African spitting cobras (*Naja* spp.), which cause systemic neurotoxicity resulting in paralysis and respiratory failure when delivered intravenously to mice, but rarely cause neurotoxicity in snakebite patients, instead typically resulting in severe local envenoming as the consequence of cytotoxicity [28].

The route of venom delivery is a major driver in the disconnect between preclinical-clinical scenarios. Snake venom is rarely delivered i.v. or i.p. during bites and thus the rapid progression to lethal venom effects modelled in mice (which can often occur within minutes), does not reflect real world envenoming where pathology is typically more progressive [29], and this might result in masking clinically relevant pathologies and the efficacy of therapy against them.

This challenge is further exacerbated when considering that snakebite therapeutics are co-incubated with snake venom ahead of murine dosing. Co-incubation promotes venom toxin-antibody binding ahead of dosing, a major factor that undoubtedly influences the ensuing measured efficacy of the treatment. Real world envenoming results in a variable time lag between venom delivery and treatment, which is often delayed by several hours due to health seeking behaviours [30]. While there has been growing momentum for new snakebite therapeutics [3] to be evaluated in ‘rescue’ models where therapy is delivered independently after venom challenge [31], almost all conventional antivenoms remain to be evaluated in this manner. Co-incubation assays are best suited for standardised potency assessment and early-stage screening in the development of antivenoms and novel therapeutics, whereas rescue models are particularly relevant for envenoming syndromes where toxicity is time-dependent and partially reversible, such as neurotoxic paralysis, as well as for systemic effects influenced by toxin distribution and clearance, including coagulopathy and myotoxicity. It has been shown that, while co-incubation assays often show complete or near-complete neutralisation under optimal conditions, rescue models reveal reduced and time-dependent efficacy owing to toxicokinetic and pharmacokinetic factors [32].

The impact of co-incubation on antivenom efficacy predictions is also problematic when considering that this model is often used as the basis for calculations of the amount of venom neutralised per millilitre (or vial) of therapeutic [33]. Such calculations are sometimes invoked to predict the likely therapeutic dose of an antivenom required to effect cure in humans in the clinical setting [34]. Because the described preclinical model of envenoming does not accurately reflect the pharmacokinetics of envenoming due to delivery via i.v. or i.p. bolus, and because venom and therapy are co-delivered, it cannot accurately reflect the pharmacokinetics of the therapy either, nor the pharmacokinetic/pharmacodynamic relationship between toxins and therapy. Thus, the lethality assay remains valuable as a comparative potency and batch-consistency tool, but it is not designed or intended to support clinical dosing predictions.

Pathology-specific assays are often more reflective of clinical pathology. However, their clinical translational value can be undermined by the way venom-induced pathology is generated. To reliably produce quantifiable necrosis within observation windows, studies frequently rely on comparatively high venom doses delivered to a single standardised site. This may be difficult to achieve with some venoms due to appearance of systemic effects (e.g., neurotoxicity). Similarly to the lethality assay, pre-incubation of therapy and venom is standard for many of these assays [5], subjecting the current pathology-specific assays to the same issues.

#### Endpoint limitations.

Although the endpoint of the lethality assay is uniform (i.e., dead or alive), the mechanism of death varies substantially depending on the venom composition. In studies using elapid venoms, such as those of cobras, kraits, mambas, and coral snakes, lethality is due to the action of neurotoxic blockade of neuromuscular transmission, resulting in progressive paralysis and respiratory failure [1,16]. In contrast, with few exceptions, viperid venoms display complex haemotoxic and cytotoxic profiles, inducing haemorrhage, coagulopathy, myonecrosis, renal injury, and cardiovascular collapse. Death therefore often arises from uncontrolled bleeding, cardiovascular shock, or acute kidney injury [1,16]. Some viperid species, such as the South American rattlesnakes, induce predominantly neurotoxic and systemic myotoxic effects [35], while Australian elapids combine neurotoxic, myotoxic, and procoagulant actions, generating overlapping mechanisms that may lead to systemic failure and lethality [36].

Because preclinical envenoming model outcomes are essentially binary (death or survival), the signs of envenoming observed in experimental animals are rarely reported [37]. The lack of granularity in these experiments is problematic because it can mask meaningful differences between therapies. For instance, two antivenoms may produce similar survival outcomes but may differ in onset, severity, or progression of any clinically relevant envenoming pathology observed, therefore having different clinical efficacy.

Pathology-specific assays are often considered to be more reflective of the clinical envenoming scenario. However, they commonly share similar endpoint constraints experienced in lethality testing. For example, determination of mouse lesion size or assessment of clotting ability is low throughput and highly prone to human subjective assessment, limiting reproducibility. Measuring the effectiveness of therapies on defibrinogenation is particularly low throughput, due to the volume of blood required for assessment, necessitating euthanasia of a mouse to obtain a single reading.

#### Limits of standardisation.

The initial global attempt to standardise how antivenoms are assessed was intended to ensure that antivenoms could be “graded easily and uniformly, in terms of their venom neutralising abilities, by different laboratories” [38]. Despite the perceived standardisation of these assays, these tests are often employed in a nonstandardised manner, with differences in animal use and procedure, providing potentially conflicting results and a barrier to comparative interpretations of resulting data and informative meta-analysis [37]. As closely as possible, experimental reporting should adhere to the ARRIVE 2.0 guidelines [39], a set of evidence-based reporting standards designed to improve the transparency, reproducibility, and quality of research involving animals, with recent venom-specific guidance on how to achieve this published [40], to enable sufficient scrutiny and reproducibility and comparison of results across laboratories.

#### Ethics and animal welfare.

These animal models carry significant ethical implications. Each LD₅₀ or ED₅₀ experiment typically requires large numbers of animals, often a minimum of 25 mice, meaning that, given the global scale of antivenom production, overall animal use is substantial. Considering that many antivenom manufacturers exist globally, this burden is further compounded by the ethical cost of animal consumption considering the severity of these assays, in which animals may experience acute, life-threatening effects for up to 24–48 h [41]. Recognising this, the WHO strongly emphasises the need to minimise pain, distress, and suffering in experimental animals [5]. In parallel, regulatory frameworks such as the EU Directive 2010/63/EU and the UK Animals (Scientific Procedures) Act (ASPA) require that animal use is scientifically justified and that the principles of the 3Rs, replacement, reduction, and refinement, are applied. However, there remain variations in standards and implementation globally, underscoring the importance of continued efforts to promote more humane and consistent approaches to toxicity testing. These limitations indicate that reform cannot rely on any single replacement assay or technical advance. Instead, progress will require a staged approach that improves existing animal models where they remain necessary, develops pathology-specific endpoints that better reflect clinical envenoming, and validates replacement methods for defined venom–antivenom contexts.

## Refining preclinical models to better reflect real world scenarios and clinical pathophysiology

To enhance the translatability of any preclinical animal models, they should (a) closely replicate the real world pathophysiology and context of the disease, and (b) incorporate endpoints that are clinically relevant indicators of therapeutic efficacy [42]. Whilst lethality is undeniably a real outcome of snakebite envenoming, it is well established that different venoms cause distinct pathologies, very broadly categorised as typically neurotoxic, haemotoxic, or cytotoxic, that may ultimately lead to death or permanent tissue damage without appropriate intervention [1].

The mechanism of lethality in standard preclinical envenoming models is often not reflective of mechanisms of lethality in the clinical scenario, which may impact on translatability [43]. Notably, target product profiles (TPPs) for antivenoms now emphasise clinical pathology-specific outcomes in addition to lethality [44]. Advances in technology now provide opportunities to refine such models, improving their resolution and relevance. Pathology-specific approaches are generally less severe, require smaller venom doses, yield richer datasets, and are more likely to translate to clinical outcomes, and thus also carry stronger ethical justification compared with lethality-based models [41].

### Innovation in preclinical models of coagulopathy

Mouse models of coagulopathy exist, although they are limited by poor resolution and species and strain specific variability and are reliant on subjective judgement. More advanced tools such as thromboelastography (TEG) and rotational thromboelastometry (ROTEM) now allow much higher resolution assessment capable of recording dynamic haemostatic changes, and are more frequently being employed in preclinical models of envenoming [45]. However, these approaches remain limited by the requirement of equipment not readily available in many quality control laboratories and large blood volumes requiring mouse euthanasia to obtain enough blood for assessment. The increasing availability of point of care devices that require substantially smaller blood volumes [46] supports the use of microsampling - the collection of small blood volumes (typically ≤50 µL) from a single animal–which can be used to measure haemostatic parameters in preclinical models. This approach offers clear 3Rs benefits, enabling reduction by allowing multiple measurements from the same animal and refinement through less invasive procedures and reduced blood loss,

### Innovation in preclinical models of tissue damage

Venom-induced cytotoxic pathology is complex and multifactorial, being induced by different toxin classes, with standard models of necrosis particularly impacted from inter-operator variability and the single timepoint readout. Recent innovations aim to address these issues; one such advance is VIDAL, a freely available machine learning–based tool designed to accurately quantify dermonecrosis in mouse models of envenoming [47]. VIDAL segments lesion images into light and dark necrotic areas and introduces the Dermonecrotic Unit (DnU), which reflects both lesion size and severity. This metric aligns closely with histopathological findings, offering faster analysis and more objective quantification of pathology.

The extreme pathology involved with cytotoxicity, although often not lethal, makes development of preclinical models of cytotoxicity ethically fraught. Substantial in-roads into treating and understanding cytotoxic envenoming pathophysiology are likely to be made by maximising the data return from such experiments. For example, the inflammatory response to venoms in humans and mice remains relatively uncharacterised. Many inflammatory markers can be rapidly read from small blood volumes using microsampling approaches in various high-throughput and widely available assays [48]. Cytotoxic pathology may also be especially amenable to emerging nonanimal or *ex vivo* models, including human tissue models, which could reduce reliance on *in vivo* studies [49].

### Innovation in preclinical models of neurotoxicity

Neurotoxicity is a major cause of snakebite mortality and is the only major pathology not to have a standardised pathology-specific model. In human snakebite victims, neurotoxicity typically presents as a descending flaccid paralysis, potentially progressing to respiratory muscle failure [50]. In contrast to coagulopathy, neurotoxic envenoming lacks routine, validated biochemical markers or diagnostic tests for diagnosis or monitoring, making serial bedside physical examination, including assessment of eye movements, ptosis, speech, and respiratory effort, essential for detecting progression towards respiratory failure [29,50].

Reproducing this progression in mice and performing such fine physical measurements would be extremely difficult and subjective. Physical assessments of neurotoxic envenoming in mice are often performed on more overt signs, such as limb paralysis. However, limb paralysis is a poor predictor of respiratory compromise [50]. Because of these limitations, more precise assessments of neurotoxic envenoming rely on *ex vivo* models, such as electrophysiological nerve-muscle preparation assays [51]. These setups demand dissection skills, highly specific apparatus (e.g., force transducers, electrical stimulators) and strict environmental control, making them technically demanding and inherently low throughput.

Because respiratory failure is the primary lethal mechanism of neurotoxic envenoming, proxies of lung function offer promise for developing neurotoxic pathology-specific models. Pulse oximetry devices are increasingly available for use in conscious mice [52]; however, this technique may not be a reliable marker of detecting early hypoxaemia caused by neurotoxicity [53]. More sensitive measures of respiratory function such as blood gas analysis may be more reliable indicators of respiratory compromise in envenoming [50]. Nevertheless, while devices for measuring these components in rodents are available, they are currently expensive, often low throughput, require arterial blood and so necessitates anesthetised mice and specialist training. However, the growing accessibility of point-of-care devices that measure physiological parameters relevant to neurotoxic envenoming, including blood gas measurements, may facilitate progress in this area in the near future.

Biochemical biomarkers may also provide useful adjunctive endpoints, particularly for venoms that cause presynaptic neurotoxicity. Although biomarkers for neurotoxicity remain underexplored in snakebite, neuronal injury markers have been widely investigated in other neurological disorders, including traumatic brain injury, multiple sclerosis, dementia, and Parkinson’s disease [54,55]. Examples include neurofilament light chain (NfL), a marker of neuro-axonal damage, and ubiquitin C-terminal hydrolase L1 (UCH-L1), a marker of acute neuronal injury. These biomarkers may be particularly useful in reduced-severity models involving predominantly presynaptic venoms, where paralysis may reflect structural damage to motor nerve terminals rather than reversible neuromuscular blockade alone.

### Syndrome-specific models for “noncanonical” envenoming patterns

Envenomings by some snakes are characterised by unique pathophysiological manifestations that can be modelled in preclinical assays. For example, the venom of certain *Daboia* spp. induces a systemic capillary leakage syndrome associated with haemoconcentration [56]. This effect has been modelled in mice by an increase in haematocrit 1 h after injection of low doses of venom [57]. Likewise, these envenomings are characterised by acute kidney injury, a leading cause of death in these cases. This effect can be monitored in rodents by quantification of the serum/plasma concentration of creatinine and urea [58]. Thus, depending on the main clinical manifestations of envenomings, a set of relevant tests, different from lethality, can be used to assess the neutralising efficacy of antivenoms.

## How applying the 3Rs can improve practical and ethical outcomes in envenoming research

The work of Russell and Burch (1959) [59] introduced the concept of the 3Rs in biomedical research and drug development and testing. This paradigm is particularly relevant in the study of venoms and antivenoms, which are heavily reliant on preclinical models that often inflict extreme pain and distress, and calls have been made to accelerate application of the 3Rs in this field [5,41].

### Refinement

Refinement aims to minimise pain, suffering, distress, and lasting harm in animals, while acknowledging that such improvements are fundamental to generating high-quality science. Whilst refinement strongly focuses on minimising pain, suffering, and distress of animals, it is often overlooked that its primary scientific value lies in producing more robust, reproducible, and clinically translatable data [60]. By limiting unnecessary severity and optimising experimental design and endpoints, refinement strengthens data robustness, reproducibility, and translational relevance, yielding clearer readouts, reduced variability, and models that better reflect clinically relevant biology [61]. Refinement is also a regulatory requirement in some jurisdictions where the most refined methods should be used, including humane endpoints.

#### Improving animal welfare in all envenoming settings.

During envenoming experiments, mice experience extreme pathologies, and therefore, it is essential to implement the use of pain relief to minimise pain and suffering. The routine use of analgesia, based on the administration of tramadol, buprenorphine, or morphine, has been established in several laboratories working with venoms and antivenoms [62]. However, despite recommendations in WHO guidelines [5], which are typically advisory rather than mandatory, many countries follow their own regulatory frameworks and, therefore, analgesia use does not appear to be widely implemented in practice. This seems to be due to concerns of analgesia potentially contributing to an increase in venom-induced pathology caused by neurotoxic venoms. Analgesia should always be employed unless experimental evidence demonstrates otherwise.

There is also space to reduce the duration of lethality assays, i.e., a refinement, which usually lasts for 24 or 48 h [5]. Several experimental studies have demonstrated that, for many venoms, there is no statistical difference in results obtained at shorter periods (6 h vs 24 h or 24 h vs 48 h) [63,64]. Instituto Clodomiro Picado has validated the use of a reduced 6 h preclinical assessment of their antivenoms, demonstrating that reduced time frame experiments can be implemented in industrial settings [9]. Importantly, this innovation must be validated for different venom/antivenom models.

#### Humane endpoints.

The use of lethality as an endpoint in preclinical models is increasingly restricted and discouraged in many jurisdictions, with a strong emphasis on replacing death with earlier humane endpoints wherever scientifically feasible [41]. This aligns with the OECD guidelines which promotes the identification of early signs of pain and distress to enable timely intervention and avoid unnecessary suffering. For studies with venoms, these include seizure, loss of self-righting reflex, and dyspnoea. Euthanising envenomed mice based on moribundity is now practiced in several academic laboratories [65–67]. One of the issues with applying currently used humane endpoints is the large degree of subjectivity and variability in pathology. There is a need to develop specific, validated, and quantifiable endpoints to enable increased confidence in implementing humane endpoints whilst not impacting the interrogation of the therapy under investigation. One such defined endpoint is reduced body temperature, which has emerged as an objective predictor of mortality in a variety of preclinical mouse models [68], and recent studies have demonstrated a strong correlation with reduced body temperature and lethality in snake venom envenomed mice [69]. Implementation of such monitoring is straightforward and affordable, utilising noninvasive handheld infrared thermometers.

### Reduction

In the context of the 3Rs, reduction refers to strategies to reduce the number of animals required in preclinical research through better designed experiments, appropriate statistical controls, and maximising the amount of information retrieved per animal [70]. Animal use can be reduced by thorough *in vitro* assessment of antivenoms prior to *in vivo* assays, to identify therapies which are unlikely to provide any protection *in vivo*. This will lead to an overall reduction in the number of animals used for preclinical testing. The use of ‘staging’ and dose-finding assays may also be considered. These approaches enable a more refined estimation of the potential range of treatment efficacy, thereby reducing the overall number of mice required by limiting experimental groups that would be uninformative for downstream statistical analysis [5].

### Replacement and new approach methodologies

Replacement refers to methods that avoid or replace the use of animals in experiments where they would otherwise have been used. This includes full replacement, in which no animals are used, as well as partial replacement approaches that replace vertebrate animals with less sentient systems, such as invertebrates (e.g., *Galleria* larvae) or early life stages of vertebrates (e.g., zebrafish embryos or chick embryos) [70]. In turn, New Approach Methodologies (NAMs) comprise a range of approaches, including *in vitro* systems, biochemical assays, and computational models, that can be used to generate data without the use of animals. These methods can support and, in some cases, enable the replacement of animal use, either individually or when applied in an integrated manner.

For many years, the argument that animals could not be replaced in preclinical antivenom testing, owing to the complex, multi‑systemic effects of venoms, has understandably supported the continued reliance on *in vivo* assays. However, advances in our understanding of venom–antivenom interactions, together with the validation of *in vitro* approaches, have made it increasingly feasible to substitute certain *in vivo* tests with well‑defined *in vitro* methods, enabling meaningful replacement of animals in specific contexts.

Besides the ethical issues of inflicting pain and distress in animals, the translatability of animal models to humans has been questioned, as evidenced by the high rate of attrition in the development of new drugs [71]. In addition, the consequences of animal testing on the mental health of people running the tests are another argument favouring the need to emphasise on Replacement [72,73].

A distinction needs to be made between the relevance of animal testing depending on the stage of antivenom development. In the early antivenom development phase, nonanimal tests aimed at evaluating the neutralisation of a variety of toxic activities besides lethality should be considered, as well as aspects dealing with pharmacokinetics/pharmacodynamics, together with innovative *in vitro* tests that correlate with *in vivo* toxicity. In contrast, once an antivenom has been developed and tested in the preclinical setting, the routine quality control of antivenom manufacture can be based on a reduced number of assays, ideally *in vitro* assays, shown to correlate with *in vivo* toxicity tests.

#### Replacement in manufacturing scenarios.

Significant efforts have been carried out for developing *in vitro* tests that correlate with *in vivo* toxicity in the assessment of venom toxicity and antivenom neutralising ability [74]. The correlation of the neutralisation of lethal activity by antivenoms and enzyme-linked immunosorbent assays (ELISAs) has been explored for a number of venoms. In some cases, a good correlation has been described, thus allowing the use of this immunoassay in antivenom potency estimation [75,76]. Notably, one manufacturer has validated ELISA in the routine quality control of antivenoms (Ian Cameron, Micropharm, personal communication). In contrast, in other venom/antivenom systems such correlation is lacking [77]. Thus, no generalisations can be made and a case-by-case analysis is necessary. Inter-laboratory validation of *in vitro* assays is required, stressing the need for international co-operation. Antivenomics represents another *in vitro* alternative based on immunoaffinity, which allows a qualitative and quantitative assessment of the venom components immuno-recognised by an antivenom [5,78]. Because of considerable snake venom variation found both inter- and intra-species [17], careful consideration should be given to ensure that the most appropriate assays and endpoints are applied for the particular venom under study. Thus, a careful case-by-case analysis is required before the introduction of new assays in the quality control of antivenoms.

**Fig 3:**
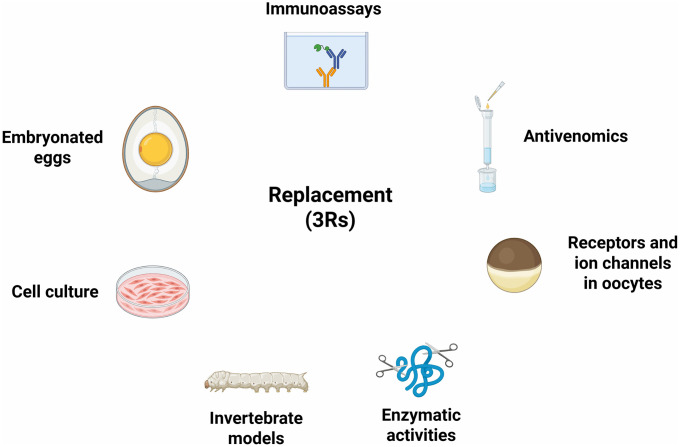
Some alternatives that might replace the mouse assays in the study of venom toxicity and antivenom neutralising efficacy. Some tests involve a full replacement of mouse models, whereas others replace mice by other animals. The selected tests must be validated on a case-by-case basis depending on the particular venom or toxic effect being assessed. Created in BioRender. Gutiérrez, J. M. (2026) BioRender.com/1btetpx.

For some venoms and antivenoms, a correlation has been described between the neutralisation of lethality and of PLA_2_ or *in vitro* procoagulant activities [79], between proteolytic and haemorrhagic activities [80], as well as between lethality and immunoassays [75,76] and agglutination [81]. The use of hen’s eggs at a developmental stage when no reflex pain arcs have yet developed has been proposed to assess venom toxicity and neutralisation by antivenoms [82,83], and zebrafish is being used to assess venom toxicity [84]. Likewise, invertebrate models, which are not protected species, such as the *Artemia salina* assay of lethality, have potential use in the assessment of antivenom potency estimation. This model has shown a good correlation with the mouse lethality assay with a variety of venoms, particularly in the assessment of antivenom neutralising ability [85]. Owing to its simplicity and low cost, it should be further explored with additional venoms and antivenoms. When addressing the introduction of novel tests in laboratories of low- and middle-income countries, the economic and technical feasibility of such innovations, such as cost and throughput, needs to be carefully considered. Figure 3 depicts some of the alternatives to test venom toxicity and antivenom neutralisation following the 3Rs principles.

#### Replacement in academic scenarios.

More complex NAMs are likely to be adopted in fundamental venom–antivenom research. Mechanism-focussed NAMs are particularly promising where the clinically dominant syndrome maps to a definable target, for example, clotting factors in VICC or acetylcholine receptor in neurotoxicity, but also when dealing with more complex pathophysiological effects, e.g., acute kidney injury. In the case of elapid snake venoms rich in post-synaptically acting neurotoxins, *in vitro* assays assessing the binding to the cholinergic receptor [86] or tests based on cells expressing the γ-subunit containing muscle-type cholinergic receptor and a fluorescent dye that reports changes in cell membrane potential [87] have been described.

Traditional cell culture methodologies have been used to assess the cytotoxicity of snake venoms and toxins, using endothelial cells [88,89], myoblasts/myotubes [89], and keratinocytes [14] as models to assess damage to the vasculature, muscle tissue, and skin, respectively. However, cell culture does not necessarily reproduce the *in vivo* scenario owing to the complexity of the action of venoms on the whole organism. Therefore, new models that ensure translatability are required. For muscle tissue, isolated skeletal muscle fibres [90] are closer to the mature muscle structure and physiology. In the case of skin necrosis induced by venoms, an organotypic model of human skin [49] and an *ex vivo* human skin model [91] have been used. Furthermore, the use of spheroids provides a three-dimensional frame to assess venom toxicity [92] and the effects on angiogenesis [93].

**Fig 4:**
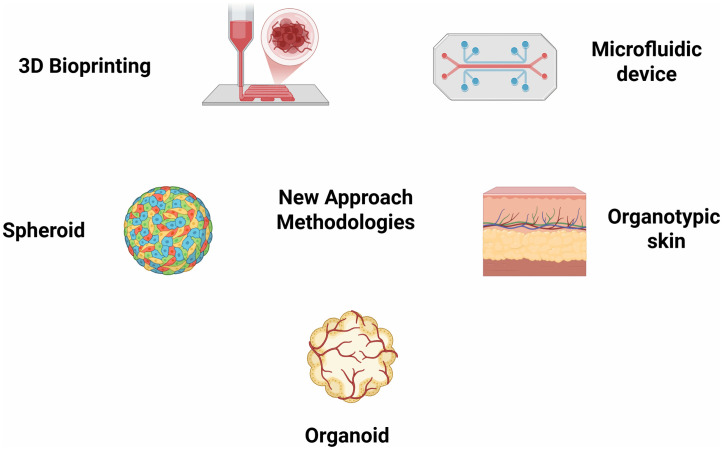
Examples of new approach methodologies that may be applied to the study of venom toxicity and antivenom neutralising efficacy, especially in the case of venom-induced effects that have a complex pathophysiology. Created in BioRender. Gutiérrez, J. M. (2026) BioRender.com/ifsheyf.

A further level of complexity is achieved by using organoids, *in vitro* three-dimensional models of organs generated from stem cells [94]. Organoids have been used in the study of toxins [95] and may become useful tools to model the effects of venoms on organs such as the kidney [96]. Organ- and organoid-on-a-chip systems are three-dimensional engineered micro- or milli-scale platforms composed of hollow, flexible polymers that support cell growth and controlled fluid flow, modelling key aspects of distinct organ physiology alongside relevant pressure and shear stress [97]. 3D bioprinting is another technology with potential application in the field of venoms. Small units of cells and extracellular matrix are dispensed to generate tissue-like structures [98]. Despite the significant potential of these methodologies, no NAM has to date been implemented for antivenom potency assessment, and further research is needed to advance their application in this area. In addition, adapting NAMs for use in quality control laboratories—particularly in low- and middle-income countries—may present important technical and economic challenges that require careful consideration. Successful uptake will also depend on broader societal and behavioural change to support acceptance and integration of these approaches. Figure 4 summarises some NAMs that could be applied to the study of venom toxicity and neutralisation by antivenoms.

## Regulatory perspectives

The increasing adoption of NAMs across regulatory frameworks reflects a global shift towards more human-relevant, mechanism-based approaches for safety and efficacy assessment. This transition is being translated into policy and practice through major initiatives, including the UK, whereby the Medicines and Healthcare products Regulatory Agency (MHRA) provides guidance supporting NAMs, including advanced *in vitro* systems, computational modelling, and organ-on-chip technologies, and EU strategies to phase out animal testing, such as the EU Commission roadmap to phase out animal testing, alongside US Food and Drug Administration (FDA). Beyond these regions, international efforts, such as the Organisation for Economic Co-operation and Development (OECD) and global partnerships, including the European Partnership for Alternative Approaches to Animal Testing (EPAA), European Medicines Agency (EMA), and United States Environmental Protection Agency (EPA), are driving a coordinated transition towards reducing and replacing animal testing. Collectively, these advances emphasise validation, harmonisation, and the integration of NAMs into regulatory decision-making, signalling a broader move towards nonanimal, human-relevant testing paradigms worldwide.

Against this evolving landscape, regulatory bodies play a central role in enabling the implementation of the 3Rs within venom and antivenom quality control and manufacturing. Current frameworks, including WHO guidelines, continue to rely heavily on *in vivo* lethality neutralisation assays, reflecting both historical precedent and the complexity of venoms and their diverse toxic effects. However, advances in the mechanistic understanding of envenoming are increasingly supporting the development of targeted *in vitro* assays that capture specific toxin activities and modes of action.

To translate these advances into regulatory practice, more open and sustained dialogue between regulators, method developers, manufacturers, quality control laboratories, and end users is essential. Such engagement will help ensure that new approaches are developed with regulatory requirements and practical applications in mind, while also enabling regulators to engage early with emerging technologies and their underlying scientific rationale. In parallel, coordinated efforts linking basic research, assay development, and regulatory evaluation remain critical. In particular, the generation of robust, context-of-use-driven evidence, together with transparent and collaborative validation studies, will be key to demonstrating the reliability, reproducibility, and predictive relevance of alternative methods. Together, these efforts will be essential to build regulatory confidence and enable the progressive integration of NAMs into antivenom quality control, ultimately supporting a transition towards more ethical, scientifically robust, and human-relevant testing strategies.

## Conclusions: A multi-stakeholder roadmap for implementing the 3Rs in antivenom development and control

**Fig 5:**
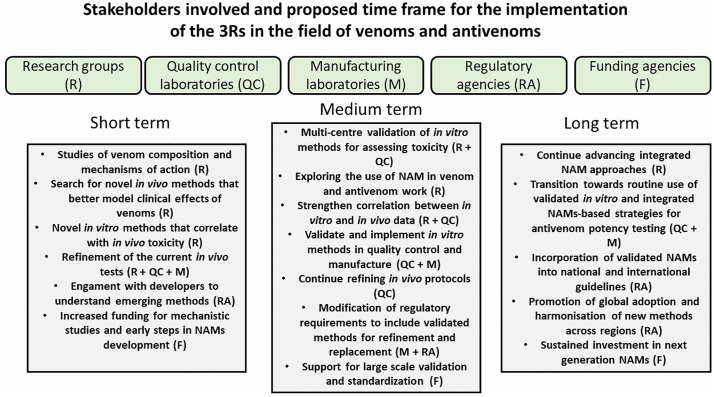
Summary of the stakeholders to be involved in a roadmap to incorporate the 3Rs paradigm in the field of venoms and antivenoms, including the main goals to be achieved at the short, medium, and long terms.

The implementation of a roadmap to align the field with the 3Rs, while improving clinical relevance, requires clearly defined roles for key stakeholders within a coordinated and time-bound framework. This process should follow a stepwise progression from knowledge generation and method development, through validation and integration, to regulatory acceptance and routine implementation. The following sections present a series of tasks proposed for each stakeholder at short-, medium-, and long-terms, and Fig 5 summarises some of the key aspects of this roadmap.

### Short-term (0–3 years): Knowledge generation and alignment

*Research groups*:

- Generate detailed information on venom composition, variability, and toxin profiles.
- Identify medically relevant toxins and assess antivenom immunoreactivity.
- Develop and explore improved *in vivo* models of envenoming where necessary, alongside early-stage *in vitro* assays correlating with *in vivo* outcomes.
- Prioritise the development of NAMs targeting specific toxin activities and pathophysiological processes, tailored case-by-case to different venoms.
- Establish close coordination with quality control and manufacturing laboratories to support rapid translation.

*Quality control and manufacturing laboratories*:

- Monitor emerging research developments and assess feasibility for routine use.
- Begin implementing refinement measures in existing *in vivo* assays (e.g., analgesia, humane endpoints, reduced assay duration).

*Regulatory agencies*:

- Engage early with developers to understand emerging methods and define context-of-use expectations.
- Encourage dialogue and provide scientific advice pathways for NAMs development.

*Funding agencies*:

- Prioritise funding for mechanistic studies and early-stage NAMs development, particularly those addressing key toxicity endpoints.

### Medium-term (3–7 years): Validation, standardisation, and early integration

*Research groups and quality control laboratories (collaborative role)*:

- Conduct multi-centre validation studies, similar to European Centre for the Validation of Alternative Methods (ECVAM), Brazilian Center for the Validation of Alternative Methods (BraCVAM), and the Interagency Coordinating Committee on the Validation of Alternative Methods (ICCVAM) based in North America, to establish reliability, reproducibility, and transferability of *in vitro* methods, especially those involving NAMs.
- Strengthen correlation between *in vitro* and *in vivo* data, demonstrating predictive relevance.
- Standardise assays, including reference materials, endpoints, and performance criteria.

*Quality control laboratories*:

- Validate and implement fit-for-purpose *in vitro* assays to complement or partially replace *in vivo* lethality tests.
- Continue refining *in vivo* protocols to minimise severity and animal use.

*Manufacturing laboratories*:

- Integrate validated alternative methods into internal quality control workflows.
- Propose evidence-based updates to testing strategies to regulatory authorities, supported by validation data.

*Regulatory agencies*:

- Maintain ongoing dialogue with stakeholders, reviewing validation data and piloting acceptance of NAMs in defined contexts.
- Enable case-by-case acceptance of alternative approaches, including combined (tiered) testing strategies.

*Funding agencies*:

- Support large-scale validation initiatives, collaborative networks, and infrastructure for standardisation.

### Long-term (7–15 years): Regulatory acceptance and full implementation

*Research groups:*

- Continue advancing integrated NAMs approaches, including multi-endpoint assays and systems-based models of envenoming.

*Quality control and manufacturing laboratories*:

- Transition towards routine use of validated *in vitro* and integrated NAMs-based strategies for potency testing.
- Limit *in vivo* lethality assays to exceptional cases where no alternative is available.

*Regulatory agencies*:

- Formally incorporate validated NAMs into national and international guidelines (e.g., WHO frameworks, International Council for Harmonisation of Technical Requirements for Pharmaceuticals for Human Use (ICH), OECD).
- Promote global harmonisation of testing strategies across regions to maximise 3Rs impact, recognising that differing regional regulatory requirements can drive duplicative testing and increase animal use. Greater alignment can help ensure that data are accepted across jurisdictions, reducing unnecessary studies.

*Funding agencies*:

- Sustain investment in next-generation NAMs (e.g., organ-on-chip, computational modelling) and continuous method improvement.

The successful implementation of this multi-stakeholder roadmap requires coordinated action across all levels, with clearly defined responsibilities and leadership, measurable outcomes, and realistic timelines. Through sustained collaboration and a strong evidence base, the field can progressively embed the 3Rs into venom and antivenom research and quality control, ultimately advancing towards more ethical, scientifically robust, and human-relevant testing paradigms.

**Box: Key learning points**

- Protocols based on animal models, especially the mouse lethality assay, have been the gold standard in the assessment of snake venom toxicity and neutralisation by antivenoms.
- The mouse lethality assays (median lethal dose and median effective dose) have been criticised on ethical and scientific grounds since they induce severe pain and distress and do not reflect the actual circumstances of snakebite envenoming.
- Alternative animal-based models are being adapted, aimed at better representing the most relevant effects induced by snake venoms in humans, while reducing animal suffering.
- Efforts are being conducted along the 3Rs paradigm (Replace, Reduce, and Refine) in animal studies with venoms and antivenoms. Emphasis should be placed on the Replace component of the 3Rs by the use of *in vitro* assays that correlate with *in vivo* animal models.
- New Approach Methodologies (NAM), i.e., *in vitro*, *in silico*, or chemical-based approaches that adequately model the effects of toxic substances as applied to humans with the goal of achieving maximal clinical mimicry, should be implemented in the study of venoms and antivenoms.

**Box: Five selected references in the field**

(1) Gutiérrez JM, R. Casewell N, Laustsen AH. Progress and challenges in the field of snakebite envenoming therapeutics. Annual Review of Pharmacology and Toxicology. 2025;65:465–485.

An overview of the three main lines of therapeutics for snakebite envenoming, including animal-derived antivenoms, recombinant antibodies, and chemical inhibitors.

(2) World Health Organization (WHO). Guidelines for the production, control, and regulation of snake antivenom immunoglobulins. Geneva, World Health Organization; 2017.

The official WHO document on various aspects of antivenom manufacture and control. It includes a section that describes the animal-based models for the preclinical evaluation of antivenom neutralising efficacy.

(3) Marriott AE, Casewell NR, Lilley E, Gutiérrez J-M, Ainsworth S. Improving *in vivo* assays in snake venom and antivenom research: a community discussion. F1000Res. 2024;13:192.

A discussion on the rodent lethality assay to assess venom toxicity and antivenom potency, emphasising on the limitations of the assay and the need to search for novel alternatives based on a better understanding of snakebite envenoming and the implementation of the 3Rs.

(4) Lauwereyns J, Bajramovic J, Bert B, Camenzind S, De Kock J, Elezović A, et al. Toward a common interpretation of the 3Rs principles in animal research. Lab Animal. 2024; 53:347–350.

An overview of the current landscape of the 3Rs paradigm as an evolving framework focussed on the continued improvement of methods aimed at better scientific outcomes and animal welfare, with emphasis in Replacement.

(5) Rial A, Morais V, Rossi S, Massaldi H. A new ELISA for determination of potency in snake antivenoms. Toxicon. 2006;48:462–466.

An example of a study that proposes the possible replacement of the *in vivo* mouse lethality assay by an *in vitro* ELISA test for the assessment of the neutralising potency of antivenoms.
